# Microglial inflammation after chronic spinal cord injury is enhanced by reactive astrocytes via the fibronectin/β1 integrin pathway

**DOI:** 10.1186/s12974-020-02059-x

**Published:** 2021-01-06

**Authors:** Shingo Yoshizaki, Tetsuya Tamaru, Masamitsu Hara, Ken Kijima, Masatake Tanaka, Dai-jiro Konno, Yoshihiro Matsumoto, Yasuharu Nakashima, Seiji Okada

**Affiliations:** 1 Department of Orthopedic Surgery, Graduate School of Medical Sciences, Kyushu University, 3-1-1 Maidashi, Higashi-ku, Fukuoka, 812-8582 Japan; 2 Department of Neuroscience & Immunology, Medical Institute of Bioregulation, Kyushu University, 3-1-1 Maidashi, Higashi-ku, Fukuoka, 812-8582 Japan

**Keywords:** Spinal cord injury, Glial scar, Reactive astrocyte, Microglia, Fibronectin

## Abstract

**Background:**

After spinal cord injury (SCI), glial scarring is mainly formed around the lesion and inhibits axon regeneration. Recently, we reported that anti-β1 integrin antibody (β1Ab) had a therapeutic effect on astrocytes by preventing the induction of glial scar formation. However, the cellular components within the glial scar are not only astrocytes but also microglia, and whether or not β1Ab treatment has any influence on microglia within the glial scar remains unclear.

**Methods:**

To evaluate the effects of β1Ab treatment on microglia within the glial scar after SCI, we applied thoracic contusion SCI to C57BL/6N mice, administered β1Ab in the sub-acute phase, and analyzed the injured spinal cords with immunohistochemistry in the chronic phase. To examine the gene expression in microglia and glial scars, we selectively collected microglia with fluorescence-activated cell sorting and isolated the glial scars using laser-captured microdissection (LMD). To examine the interaction between microglia and astrocytes within the glial scar, we stimulated BV-2 microglia with conditioned medium of reactive astrocytes (RACM) in vitro, and the gene expression of TNFα (pro-inflammatory M1 marker) was analyzed via quantitative polymerase chain reaction. We also isolated both naïve astrocytes (NAs) and reactive astrocytes (RAs) with LMD and examined their expression of the ligands for β1 integrin receptors. Statistical analyses were performed using Wilcoxon’s rank-sum test.

**Results:**

After performing β1Ab treatment, the microglia were scattered within the glial scar and the expression of TNFα in both the microglia and the glial scar were significantly suppressed after SCI. This in vivo alteration was attributed to fibronectin, a ligand of β1 integrin receptors. Furthermore, the microglial expression of TNFα was shown to be regulated by RACM as well as fibronectin in vitro. We also confirmed that fibronectin was secreted by RAs both in vitro and in vivo. These results highlighted the interaction mediated by fibronectin between RAs and microglia within the glial scar.

**Conclusion:**

Microglial inflammation was enhanced by RAs via the fibronectin/β1 integrin pathway within the glial scar after SCI. Our results suggested that β1Ab administration had therapeutic potential for ameliorating both glial scar formation and persistent neuroinflammation in the chronic phase after SCI.

**Supplementary Information:**

The online version contains supplementary material available at 10.1186/s12974-020-02059-x.

## Background

Spinal cord injury (SCI) is a devastating trauma accompanied by persistent, severe motor and sensory dysfunction [1], and almost no evidence-based therapies for chronic SCI patients have been established [2]. The main reasons why chronic SCI treatments are ineffective are suggested to be (1) the glial scar, which hinders axonal regeneration through physical and chemical barriers [3], and (2) persistent neuroinflammation, which cause SCI lesions to become refractory to treatment [4]. To modulate these inhibitory factors in the injured spinal cord, including glial scars, many studies have focused on the role of inflammatory cells and astrocytes, which are the major component of lesional scars [3, 5, 6]. However, in addition to astrocytes, the glial scar also includes microglia as a cellular component [7, 8], and the pathological roles of microglia in the glial scar are poorly understood.

Microglia are the resident immune cells in the central nervous system (CNS) and can be polarized to distinct functional phenotypes: the M1- and M2-phenotypes. Although these phenotype classifications are now recognized as oversimplified, the M1-phenotype is generally defined as pro-inflammatory and neurotoxic, whereas the M2-phenotype is considered to be anti-inflammatory, immunomodulatory, and neuroprotective [9, 10]. In our previous reports, we demonstrated that the number of microglia as well as the mRNA expression of the pro-inflammatory cytokine TNFα remained increased until the chronic phase after SCI [11]. Given that TNFα is a representative pro-inflammatory marker and that the microglial expression of TNFα reportedly converts the polarization of astrocytes to the neurotoxic phenotype [12], microglia seem to be strongly associated with chronic neuroinflammation after SCI [13]. As such, clarifying the role of microglial cells as well as their interaction with astrocytes within the glial scars would contribute to a better understanding of the pathophysiology of chronic SCI [14].

Recently, we reported that the interaction between astrocytic β1 integrin receptor (β1R) and collagen was a trigger of glial scar formation and that the administration of anti-β1 integrin antibody (β1Ab) in the sub-acute phase successfully prevented glial scar formation and enhanced axonal regeneration [5]. However, β1Rs are expressed on not only astrocytes but also microglia in the CNS [15]. In addition, β1Rs can bind with not only collagen but also other extracellular matrices (ECMs), such as laminin and fibronectin [16]. Considering that ECMs play crucial roles in microglial activation and polarization, we speculated that the therapeutic effects of β1Ab on SCI pathology might be due to not only the attenuation of glial scar but also the blockade of microglial interaction with ECMs.

In this study, we examined the glial scar pathology, including the microglial activation and polarization after SCI. As a result, we demonstrated for the first time that the interaction between astrocytes and microglia was mediated by fibronectin. β1Ab administration had a blocking effect on the intercellular interaction, which significantly suppressed chronic inflammation within the glial scar. Our results suggest that β1Ab administration has therapeutic potential for ameliorating both glial scar formation and persistent neuroinflammation in the chronic phase after SCI.

## Methods

### Mice

All study protocols involving mice were approved by the Committee of Ethics on Animal Experimentation of our institution (A30-199-0) and conducted in accordance with the National Institutes of Health guidelines for the care and use of animals. We used 8-week-old female C57BL/6N mice (body weight = 19–21 g, Japan SLC, Japan). All mice were housed in a temperature- and humidity-controlled environment on a 12-h light-dark cycle, with food/water available ad libitum. All efforts were made to reduce the number of animals used in the experiments and to minimize their suffering. In this study, we used 94 mice in total.

### Contusion SCI model

The mice were anesthetized with an intraperitoneal injection of mixed anesthesia using medetomidine hydrochloride (0.3 mg/kg), midazolam (4 mg/kg), and butorphanol tartrate (5 mg/kg) and were subjected to contusion injury (70 kilodynes) at the 9th thoracic level using an Infinite Horizons Impactor (Precision Systems Instrumentation, Lexington, KY, USA) as previously described [17]. After injury, the overlying muscles were sutured, and the skin was closed with wound clips. During the period of recovery from anesthesia, the animals were placed in a temperature-controlled chamber until thermoregulation was reestablished.

### Intralesional administration of β1Ab

After re-anesthesia and exposure of the dorsal dura matter, a glass tip was inserted 2 mm rostrally and caudally from the epicenter of the injured spinal cord, and 2 μl of β1Ab (Purified NA/LE Hamster Anti-Rat CD29, clone: Ha2/5; BD Pharmingen, San Diego, CA, USA) was administered at 0.5 μl/min using a stereotaxic injector (KDS 310; Muromachi Kikai Co., Ltd., Tokyo, Japan) every 2 days from 9 to 13 days post-injury (dpi). Control mice were given an equivalent amount of control antibody (Purified NA/LE Hamster IgM, λ1, Isotype Control; BD Pharmingen) at 9, 11, and 13 dpi. To prevent the backflow, the needle was kept in place for 2 min after injection, as previously described [5].

### Immunohistochemistry (IHC) analyses

Mice were re-anesthetized and transcardially perfused with normal saline, followed by 4% paraformaldehyde in 0.1 M phosphate-buffered saline (PBS). The spinal cord was removed and immersed in the same fixative at 4 °C for 24 h. A spinal segment centered over the lesion epicenter was transferred into 10% sucrose in PBS for 24 h and 30% sucrose in PBS for 24 h and embedded in O.C.T. compound. The embedded tissue was immediately frozen in liquid nitrogen and stored at − 30 °C until use.

Frozen sections were cut with a cryostat in the sagittal or axial plane at 16 μm and mounted onto glass slides as previously described [17]. For immunofluorescence staining, spinal cord sections were permeabilized with 0.01% Triton X-100 and 10% normal goat serum in PBS at pH 7.4 for 60 min. The sections were then stained with primary antibodies against GFAP (1:200; astrocyte marker, rat; Invitrogen, Carlsbad, CA, USA), TMEM119 (1:200; microglia marker, rabbit; Abcam, Cambridge, MA, USA), ColIα1 (1:200; mouse; Sigma-Aldrich, St. Louis, MO, USA), CD11b (1:200; rat; AbD serotec, Kidlington, UK), Fibronectin (1:200; rabbit; Dako Corp., Carpinteria, CA, USA), and Laminin (1:200; rabbit; Sigma-Aldrich). The sections were then incubated with Alexa Fluor-conjugated secondary antibodies (1:200; Invitrogen). Nuclear counterstaining was performed using Hoechst 33342 (1:1000; Invitrogen). All images were captured using a BZ-X700 digital microscope system (Keyence Japan, Osaka, Japan) or epifluorescence microscope equipped with a digital camera (BX51; Olympus, Tokyo, Japan). To evaluate the distribution of apoptotic cells in each injured spinal cord, a terminal deoxynucleotidyl transferase-mediated dUTP nick-end labeling (TUNEL) assay was performed using an ApopTag red in situ kit (Chemicon, Temecula, CA, USA) as previously described [17]. To count and compare the number of apoptotic cells, the region of the glial scar (densely overlapped GFAP-positive peri-lesional area, approximately 200 μm wide) in 5 sagittal sections with intervals of 350 μm was analyzed with the Image J (http://rsb.info.nih.gov/ij/).

### Analyses of the locomotor function

The motor function of the paralyzed hindpaws was evaluated with a locomotor open field rating scale on the Basso Mouse Scale (BMS). Each mouse was assessed at 1, 4, 7, 14, 21, 28, 35, and 42 dpi. A team of three independent examiners evaluated each animal for 4 min and assigned an operationally defined score to each hindpaw. The BMS score at 1 dpi was 0 or 1 in all mice. Every test was performed in a double-blinded fashion as previously described [17].

### Laser-captured microdissection (LMD)

Fresh injured spinal cords were immediately frozen in dry ice/hexane and stored in a deep freezer at − 80 °C, as previously described [5, 18]. The tissues were sectioned into 16-μm-thick slices using a cryostat at − 20 °C and mounted on polyethylene naphthalate membrane slides. The tissues were cut at 16 μm using a cryostat at − 20 °C and mounted on PEN membrane slides. The sections were then fixed in ice-cold acetone for 2 min and stained with the antibody against GFAP (1:50; rat; Invitrogen, Carlsbad, CA, USA) for 5 min. After definition of the GFAP-negative area as the lesion epicenter, the region of the glial scar (densely overlapped GFAP-positive peri-lesional area, approximately 200 μm wide in both control Ab- and β1Ab-treated mice), and naïve (NA, morphologically identical to resident astrocytes) or reactive (RA, hypertrophic morphology with extended processes) GFAP-positive astrocytes were dissected with an LMD 6500 system (Leica Microsystems, Wetzlar, Germany) and transferred by gravity into a microcentrifuge tube cap placed directly beneath the section. The tube cap was filled with 75 μl of buffer RLT (Qiagen, Hilden, Germany). For each sample, 20 glial scars or 500 astrocytes were dissected from each spinal cord. Although NA-like cells were seen in the peri-lesional area of β1Ab-treated mice, we were unable to histologically distinguish the NA-like cells from intact NAs. Therefore, we selectively sorted NAs and RAs only from control Ab-treated mice using LMD.

### Fluorescence-activated cell sorting (FACS)

The spinal cords (6.0 mm in length, centered around the lesion, including both the lesion epicenter and border/glial scars) were dissociated in collagenase type I (Invitrogen) and stained with the following antibodies: PECy7-conjugated CD45 (1:10), fluorescein isothiocyanate-conjugated CD11b (1:10), and PE-conjugated Gr-1 (1:50). All conjugated antibodies were purchased from eBioscience (San Diego, CA, USA). Each sample was analyzed using a FACSAria II flow cytometer and the FACSDiva software program (BD Biosciences). CD11b^high^/Gr-1^neta-int^/CD45^int^ population was isolated as the lesional microglia and subjected to RNA extraction as previously described [19].

### Quantitative real-time polymerase chain reaction (qPCR)

Total RNA was isolated from the lesional microglia with FACS or from the glial scars and astrocytes with LMD using the RNeasy Micro Kit (Qiagen) as previously described [5, 18]. For the complementary DNA (cDNA) synthesis, a reverse transcription reaction was performed using PrimeScript Reverse Transcriptase (TaKaRa, Tokyo, Japan). qPCR was performed using primers specific to the genes of interest (Table 1) and SYBR Premix Dimer Eraser (TaKaRa). The data were normalized to the expression of glyceraldehyde-3-phosphate dehydrogenase. Using one cDNA sample, we can examine the mRNA expression of various factors listed in Table 1. The term ‘*n*’ means the number of samples pooled from different mice in the same group.

**Table 1:** Primers used in the qPCR analyses

Gene	Forward primer	Reverse primer
Gapgh	5′-GACTTCAACAGCAACTCCCACTCT-3′	5′-GGTTTCTTACTCCTTGGAGGCCAT-3′
Gfap	5′-TGTACTAACAGAGCGAGCCTATGC-3′	5′-GGGACTTGCTGCCTTTAACATTGG-3′
Col1α1	5′-TGGTCACGTTCAGTTGGTCAAAGG-3′	5′-AACCCTGGAAACAGACGAACAACC-3′
Gap43	5′-GACAGGATGAGGGTAAAGAAGACC-3′	5′-AGAGAGAGAGGGCTCATAGGTAGG-3′
Tnf	5′-TGTGAAAACGGAGCTGAGCTGTCC-3′	5′-GGTTCAGTGATGTAGCGACAGCCT-3′
Msr1	5′-TGGAACACATGAAGGACATGGAGG-3′	5′-AATTCCCATGTTCCTGGACTGACG-3′
Mrc1	5′-ATGTGTCGTGATCGCAGAATTGTG-3′	5′-GGTGTATAACATATCTCTGGGAGG-3′
Ccl1	5′-TGTTACAGAAAGATGGGCTCCTCC-3′	5′-ACTGAGGGAAACTGCAGTCTCTGG-3′
Ccl8	5′-AGAAGCTGACTGGGCCAGATAAGG-3′	5′-ACTCACTGACCCACTTCTGTGTGG-3′
Ccl17	5′-ATGTGAAGAAGGCCATCAGATTGG-3′	5′-TTCGCCTGTAGTGCATAAGAGTCC-3′
Ccl22	5′-AAACTTCAGACTTCCTTGGCCTCC-3′	5′-GAAATCTGATTCTGAGCCTGCTCC-3′
Ccl24	5′-GCAGAAACGGAGGATTTCAACAGC-3′	5′-CTTCTTAGGCATGCACCATCATGC-3′
Cxcl1	5′-GGGAGGCTGTGTTTGTATGTCTTG-3′	5′-CGAGACCAGGAGAAACAGGGTTAA-3′
Cxcl2	5′-CGGATGGCTTTCATGGAAGGAGTG-3′	5′-GCTAAGCAAGGCACTGTGCCTTAC-3′
Cxcl3	5′-TAACAACTCCTGAGAGTTCATACC-3′	5′-CCTTCAAGTTAAGAATAGGCTTGG-3′
Cxcl9	5′-GGAAGCAGTAAATTCTGCGAGTGG-3′	5′-GCACAAAAACCACCTGCATGCAGF-3′
Cxcl10	5′-GTCCTGAGACAAAAGTAACTGCCG-3′	5′-AACTTAGAACTGACGAGCCTGAGC-3′
Cxcl11	5′-CTGTGAATGAATGGTAGGGATGGC-3′	5′-CTGTGAATGAATGGTAGGGATGGC-3′
Cxcl13	5′-ATACCCAACCCACATCCTTGTTCC-3′	5′-CGCAAACCTCTTGTTAGTACGAGC-3′
Il1b	5′-GGGCTGGACTGTTTCTAATGCCTT-3′	5′-CCATCAGAGGCAAGGAGGAAAACA-3′
Il6	5′-CCACAGTGAGGAATGTCCACAAAC-3′	5′-GCTCTCCTAACAGATAAGCTGGAG-3′
Il10	5′-GCTCCAAGACCAAGGTGTCTACAA-3′	5′-CCGTTAGCTAAGATCCCTGGATCA-3′
Il12b	5′-CAGAGCCAGGGAGCTAATGTATGC-3′	5′-ATTTGCATAATAGGGCCTGGTCCC-3′
Il15	5′-GTCCAAATGTTCATCAACACGTCC-3′	5′-TGATCCAAGTGGCTCATTATCTCC-3′
Il23a	5′-AGAATAAAGTCTCGAGCCCTTGGC-3′	5′-TACAGATAATGGCCAAAGCCTGGG-3′
Tgfb1	5′-GTGACAGCAAAGATAACAAACTCC-3′	5′-GAGCTGAAGCAATAGTTGGTATCC-3′
Arg1	5′-TGTATCCCAGCAGTTCCTTTCTGG-3′	5′-ACCTCTCTGGATACCTTTGCTTCC-3′
Igf1	5′-AACTGTCTGGGCCTAAAAGCAAGC-3′	5′-AACAGAGGAGACTTTGCTGTGAGC-3′
Chil3	5′-ATACACAGTGCAAGTTGCAAGGGC-3′	5′-GCTGGTACAGCAGACAAGACATCC-3′
Pdgfa	5′-AGACAGATGTGAGGTGAGATGAGC-3′	5′-ACGGAGGAGAACAAAGACCGCACG-3′
Pdgfb	5′-TACCTCCACTCTGTGTCTTCTTCC-3′	5′-CATCCCATTACAACCTTGCTCACC-3′
Fcgr3	5′-CTCACAAACAAGATGCCTACTGCC-3′	5′-GGATAACAGCTATGCCATCAACCC-3′
Fcgr2b	5′-GTCACTTCTGTGAGTCCTGAAACC-3′	5′-TATAAGCAGTTCCCACGTTGCTGC-3′
CD86	5′-GAACCATCTCTAGATCCAAGAGCC-3′	5′-TTGGCTGTCTTATCCTTGCACAGC-3′
CD163	5′-AGCTAAATGGAACAAGAGCCCAGG-3′	5′-GACCCTATTGCGAACAAGCAAACC-3′
Fn1	5′-TTTACAGCTTCTCCAAGCATCGCC-3′	5′-TCCCTATTGATCCCAGACCAAACC-3′
Fn1 transcript variant 1	5′-ACGGTTTCCCATTACGCCAT-3′	5′-TCATCCGCTGGCCATTTTCT-3′
Fn1 transcript variant 2	5′-ATGAGAAGCCTGGATCCCCT-3′	5′-GGAAGGGTAACCAGTTGGGG-3′
Fn1 transcript variant 3	5′-ATGAGAAGCCTGGATCCCCT-3′	5′-TGTCCGCCTAAAGCCATGTT-3′
Fn1 transcript variant 4	5′-ACGGTTTCCCATTACGCCAT-3′	5′-TCATCCGCTGGCCATTTTCT-3′
Fn1 transcript variant 5	5′-GACCCTTACACGGTTTCCCA-3′	5′-TCATCCGCTGGCCATTTTCT-3′
Fn1 transcript variant 6	5′-ATGAGAAGCCTGGATCCCCT-3′	5′-GAGAGCTTCCTGTCCTGTCT-3′
Fn1 transcript variant 7	5′-GACCCTTACACGGTTTCCCA-3′	5′-TCATCCGCTGGCCATTTTCT-3′
Lamb2	5′-TCGTTCTCCTCATATGTGCCCTCC-3′	5′-TTGAAAGCTCTTGCTAGCCAGGAG-3′

### Preparation of conditioned medium of RAs (RACM)

Purified primary astrocyte cultures were prepared from C57BL/6N mice as previously described [5]. In brief, after the removal of the meninges, postnatal day 3 (P3) mouse brain tissues were minced and incubated in a rocking water bath at 37 °C for 30 min in Dulbecco’s modified Eagle’s medium (DMEM; Invitrogen) in the presence of 300 g/mL DNase I (Sigma-Aldrich) and 0.25% trypsin (Sigma-Aldrich). Enzyme-digested dissociated cells were triturated with 0.25% fetal bovine serum (FBS), washed, and centrifuged at 300×*g* for 5 min. The pellet was re-suspended in DMEM, passed through a 30-μm nylon mesh, washed, and centrifuged at 300×*g* for 5 min. Following dilution with astrocyte-specific medium (DMEM containing 10% FBS, 0.2 mML-glutamine, and 1% penicillin-streptomycin), the cells were plated on poly-l-lysine-coated culture dishes at 1.0 × 10^5^ cells/cm^2^ and allowed to adhere for 1 day in a humidified CO_2_ incubator at 37 °C. Next, non-adherent cells were removed, and fresh astrocyte-specific medium was added. Adherent cells were maintained in astrocyte-specific medium for 7 days with medium changed every 2 to 3 days. For passage, monolayers were rinsed with PBS and then dislodged by trypsinization (0.25% trypsin and 0.02% ethylene diamine tetra acetic acid) for 3 min at 37 °C and plated on poly-l-lysine-coated dishes at 5.0 × 10^4^ cells/cm^2^. Passaged astrocyte cultures between 3 and 5 weeks in vitro were used throughout, unless otherwise specified. Primary astrocyte cultures were thoroughly agitated in an orbital incubator shaker at 350 rpm and 37 °C for 12 h on 7 days after their establishment. Immediately after agitation, all cells suspended in the culture medium were discarded, and attached cells were sub-cultured in astrocyte-specific medium and stimulated with 50 ng/mL IL-6 (R&D Systems Inc., Minneapolis, MN, USA) and 200 ng/mL soluble IL-6 receptor (R&D Systems Inc.) as previously described [5]. After incubation at 37 °C for 2 h, the supernatant was isolated and ultrafiltrated with Amicon Ultra-4 (Centrifugal Filter Units, 50 kDa [capturing protein fragments from 100 to 200 kDa]; Merck Millipore, Billerica, MA, USA), which we used as RACM in our in vitro stimulation experiments. We can ignore the residual effects of IL-6/IL-6R in the RACM because the molecular mass of IL-6/IL-6R is 22–28/80 kD, neither of which is captured by Amicon Ultra-4 (capturing protein fragments from 100–200 kD).

### BV2 cell line culture stimulation with RACM and fibronectin in vitro

The BV-2 murine microglial cell line (kindly provided by Dr. Biber K., Department of Medical Physiology, University Medical Center Groningen, University of Groningen) was cultured in DMEM with 5% FBS, 2 mML-glutamine, and 1% penicillin-streptomycin as previously described [17]. Thirty minutes before the RACM stimulation experiments, either β1Ab (Purified NA/LE Hamster Anti-Rat CD29, clone: Ha2/5; BD Pharmingen) or control Ab (30 μg/ml, Purified NA/LE Hamster IgM, λ1, Isotype Control; BD Pharmingen) was added. To examine the microglial response induced by humoral factors released by RA after SCI, the floating BV-2 cells were collected after incubation with a dilution series of the ultrafiltrated RACM or fibronectin (purified human fibronectin, alpha-chymotriptic fragment 120 kDa [cell attachment factor]; Merck Millipore) for 48 h. The supernatant including BV-2 cells was then centrifuged, and the pellet was isolated as the RACM-stimulated BV-2 cells, of which 1 × 10^5^ cells were subjected to qPCR.

### Statistical analyses

Wilcoxon’s rank-sum test was used to compare the median values of the qPCR data as well as the microglial cell count. All tests were 2-sided, and the level of significance was set at 0.05. The values for groups were presented as the average ± standard error of the mean (SEM). Statistical variance was also assessed by the *F* value. All statistical analyses were carried out using the JMP software program (version 13; SAS Institute, Inc., Cary, NC, USA).

## Results

### Administration of β1Ab changed the microglial distribution and inflammatory response within the glial scar after SCI

**Fig. 1:**
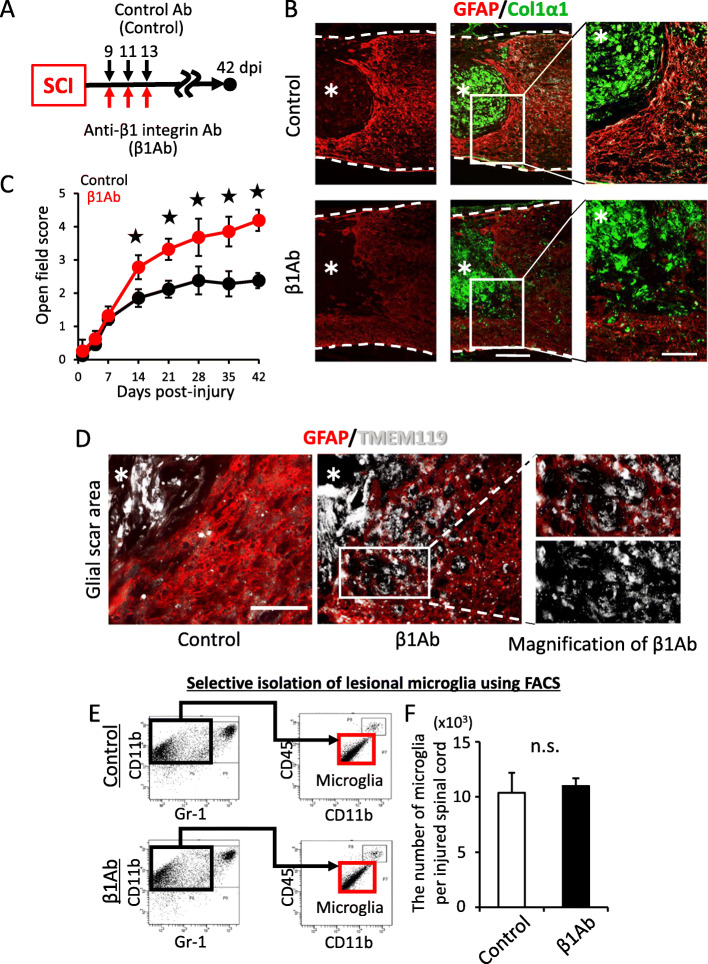
The administration of anti-β1 integrin antibody to injured spinal cord in the sub-acute phase suppressed the glial scar formation in the chronic phase, leading to changes in the microglial distribution within the glial scar. **a** Time schedule of our in vivo experiments. Injured mice were intralesionally administered anti-β1 integrin antibody or control antibody at 9, 11, and 13 days post-injury (dpi). At 42 dpi, the injured spinal cord was analyzed. **b** Sagittal sections of the chronically injured spinal cords. Magnification of the inset is shown in the right figure. Asterisk indicates the lesion epicenter. GFAP, red; ColIαI, green. Scale bars, 250 μm on the left and 100 μm on the right. **c** Time course of the Basso Mouse Scale score after SCI. Error bar indicates mean ± SEM. Star indicates statistical significance (*p* < 0.05). A two-way repeated-measures ANOVA with the Tukey-Kramer post hoc test. *n* = 14 mice per group. **d** Peri-lesional glial scar of the chronically injured spinal cord. Microglia are mainly the lesion epicenter. β1Ab administration changed the microglial distribution within glial scars. Asterisk indicates the lesion epicenter. GFAP, red, TMEM119, white. Scale bar, 200 μm. **e** Gating strategy of fluorescence-activated cell sorting for selective isolation of microglia from injured spinal cord. Red-boxed population, Gr-1^nega-int^/CD11b^high^/CD45^int^, indicates microglial population. **f** The number of resident microglia in the injured spinal cord counted by FACS. Error bar indicates mean ± SEM. n.s. indicates not significant. Wilcoxon’s rank-sum test. *n* = 3 per each group, triplicate. *F* = 0.266

We previously reported that administration of β1Ab for sub-acute SCI suppressed astrocytic phenotype changes as well as glial scar formation [5]. In the present study, we followed this protocol (Fig. 1a, Additional file 1) and focused on the microenvironmental changes other than changes to astrocytes induced by β1Ab administration. As previously reported [5], we found that a dense layer of astrocytic glial scarring was observed around the lesion area in the control group, whereas this scarring was significantly attenuated in the β1Ab-treated group (Fig. 1b, Additional file 2) [5]. We also confirmed that the open field motor score was significantly higher in the β1Ab-treated group than in the control group at 42 dpi (Fig. 1c).

In addition, we found that the microglial distribution around the lesion was significantly different between the β1Ab-treated group and the control group in immunohistochemical analyses (Fig. 1d). Within the GFAP-positive astrocytic scar area, a small number of TMEM119-positive microglial cells were observed compared to the lesion core in the control groups, whereas microglia were located evenly between the lesion core and astrocytic scar area in the β1Ab-treated group (Fig. 1d). Although there is a possibility that β1Ab administration may have attenuated the inflammatory response and altered the microglial activation, fluorescent-activated cell sorting (FACS) analyses revealed the number of microglia to be comparable between the two groups (Fig. 1e, f). These results suggested that β1Ab administration not only modulated glial scar formation but also altered the microenvironment of chronically injured spinal cords including the distribution of microglia.

### Administration of β1Ab significantly changed the expression of cytokines by both chronic glial scars and lesional microglia

**Fig. 2:**
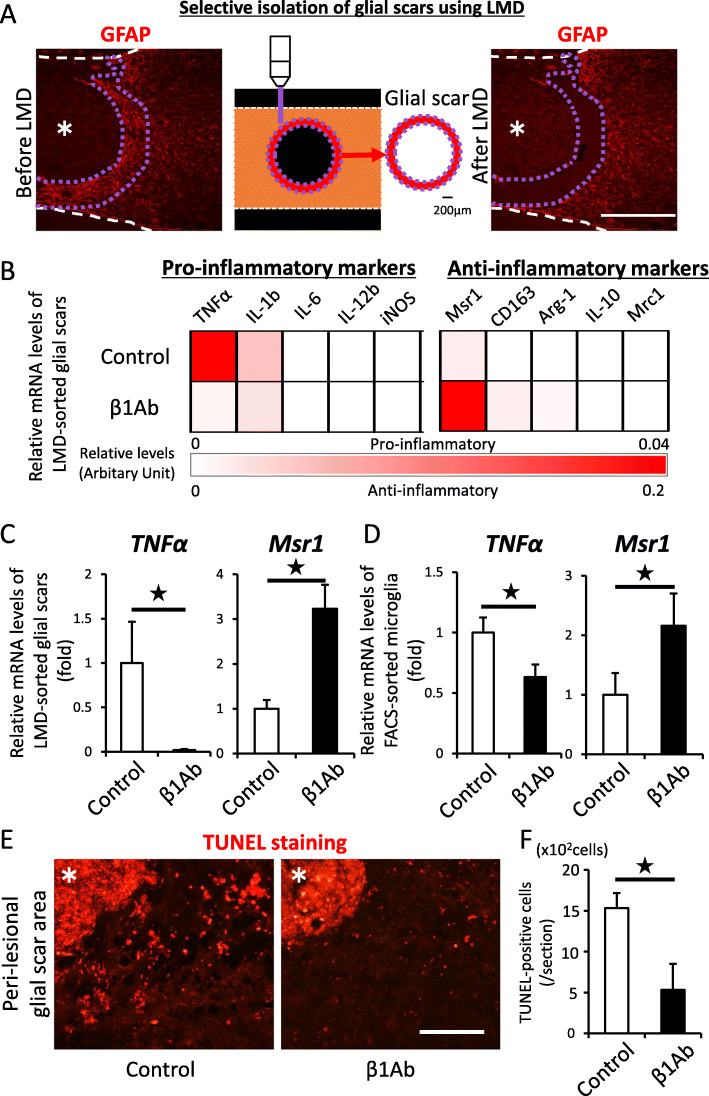
The administration of anti-β1 integrin antibody to injured spinal cord in the sub-acute phase suppressed microglial inflammation within the glial scar in the chronic phase after SCI. **a** Sagittal section before and after selective isolation of the glial scars using a laser-captured microdissection system. Purple dots indicate the cutting line of peri-lesional area. The width of the glial scar area was set at approximately 200 μm. Asterisk indicates the lesion epicenter. GFAP, red. Scale bar, 500 μm. **b** The heatmap indicates the mRNA expression profile of representative pro- and anti-inflammatory markers. The mRNA expression of TNFα and Msr1 was markedly different between the control- and β1Ab-treated groups. *n* = 4 per each group, duplicate. **c** Analyses of the mRNA expression of the glial scars by qPCR. Error bar indicates mean ± SEM. Star indicates statistical significance (*p* < 0.05). Wilcoxon’s rank-sum test. *n* = 8 per each group. TNFα: *F* = 0.0002, Msr1: *F* = 0.0201. **d** Analyses of the TNFα and Msr1 mRNA expression of microglia by qPCR. Error bar indicates mean ± SEM. Star indicates statistical significance (*p* < 0.05). Wilcoxon’s rank-sum test. *n* = 4 per each group. TNFα: *F* = 0.0365, Msr1: *F* = 0.0427. **e** TUNEL-stained sections of chronically injured spinal cords. Asterisk indicates the lesion epicenter. TUNEL-positive, red. Scale bar, 200 μm. **f** The number of TUNEL-positive apoptotic cells within glial scars in the injured spinal cord counted by the Image J software program. Error bar indicates mean ± SEM. Star indicates statistical significance (*p* < 0.05). Wilcoxon’s rank-sum test. *n* = 5 per each group, triplicate. *F* = 0.0274

To examine the influence of the altered spatial distribution of microglia within the glial scar area, we selectively isolated the toroidal region of the glial scar (peri-lesional area) from sagittal sections using LMD (Fig. 2a, approximately 200 μm wide) and performed gene expression analyses of cytokines (Fig. 2b, c, Additional file 3, 4). Because microglia are associated with inflammation, we initially speculated the peri-lesional inflammation was enhanced due to the altered distribution of microglia in the β1Ab-treated group. However, contrary to our expectations, the TNFα expression around the lesion area was significantly lower in the β1Ab-treated group than in the control group (Fig. 2b, c). Furthermore, the gene expression of macrophage scavenger receptor 1 (Msr1), which is involved in the regulation of anti-inflammatory process, was significantly upregulated in the β1-treated group (Fig. 2b, c). Given that TNFα and Msr1 are representative markers of the pro-inflammatory M1 and anti-inflammatory M2 microglia, respectively, this result suggested that β1Ab administration might affect the microglial inflammation within the glial scars.

To verify this, we directly isolated microglia from injured spinal cords using FACS and performed gene expression analyses. Consistent with the results of the glial scar area, the TNFα expression was significantly downregulated, whereas the Msr1 expression was significantly upregulated in the microglia of the β1Ab-treated group compared to those of the control group (Fig. 2d). Since the TNFα expression in microglial cells was significantly associated with lesional apoptosis [19], we performed TUNEL staining and found that the number of TUNEL-positive apoptotic cells in the peri-lesional glial scar area was significantly decreased in the β1Ab-treated group (Fig. 2e, f). These results suggest that the sub-acute administration of β1Ab significantly altered the spatial distribution of microglia and suppressed their pro-inflammatory reactions.

### Promotion of microglial inflammation by RACM

**Fig. 3:**
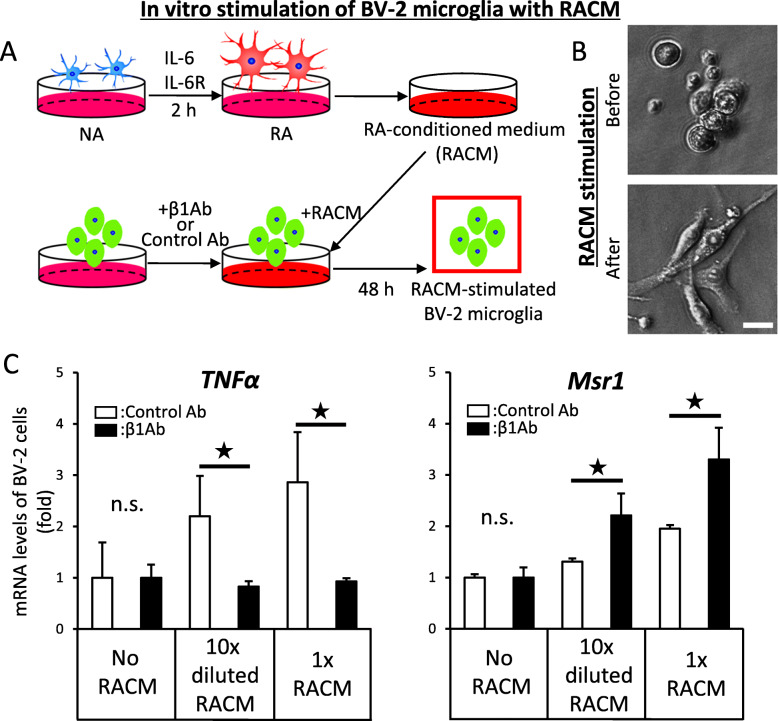
The administration of anti-β1 integrin antibody significantly suppressed pro-inflammatory polarization of BV-2 microglial cells mediated by fibronectin as well as conditioned medium of reactive astrocytes in vitro. **a** Schematic illustration of our in vitro experiments. At 2 h after stimulation of primary astrocyte culture, the conditioned medium (RACM) was collected, concentrated, and incubated with BV-2 microglia. BV-2 cells were subjected to analyses at 48 h after the stimulation. **b** Phase contrast images of BV-2 cells before and after RACM stimulation. **c** Analyses of the mRNA expression of BV-2 cells after pre-treatment with control antibody or anti-β1 integrin antibody and RACM stimulation by qPCR. The concentration of 1× RACM was 10 times denser than 10×-diluted RACM. Error bar indicates mean ± SEM. Star indicates statistical significance (*p* < 0.05). Wilcoxon’s rank-sum test. *n* = 3 per each group, triplicate. TNFα: *F* = 0.306, 0.0256, and 0.0014, respectively. Msr1: *F* = 0.872, 0.047, and 0.032, respectively

Given the recent finding that the lesional microenvironment is crucial for astrocytic polarization [5], we next examined the effects of β1Ab administration on microglial inflammation in vitro. After SCI, in vivo NAs are activated and transformed to RAs, expressing various proteins. RACM was the ultrafiltrated supernatant of in vitro RAs incubated with IL-6/IL-6R for 2 h and included humoral factors released by RAs. To mimic the in vivo microenvironment of sub-acute SCI in vitro, we cultured BV-2 microglia and performed stimulation with RACM, evaluating the changes in the morphology and mRNA expression of microglia were analyzed 48 h later (Fig. 3a, Additional file 5).

The morphology of BV-2 cells was significantly changed from round to spindle-shaped by RACM stimulation (Fig. 3b), suggesting that BV-2 microglia were activated by RACM [19]. Consistent with this finding, the TNFα mRNA expression was upregulated in a dose-dependent manner by serial-diluted RACM (Fig. 3c), suggesting that certain factors secreted by RAs activated microglia to a pro-inflammatory condition.

However, when BV-2 microglia were pre-treated with β1Ab, the upregulation of the TNFα mRNA expression was significantly suppressed, while the Msr1 mRNA expression was significantly upregulated, even with RACM stimulation (Fig. 3c). These results suggest that β1Ab can interact with certain ligand factors in RACM, and thereby suppress the microglial inflammation, while promoting the resolution of chronic inflammation after SCI.

### Fibronectin is expressed by reactive astrocytes and associated with microglial inflammation after SCI

**Fig. 4:**
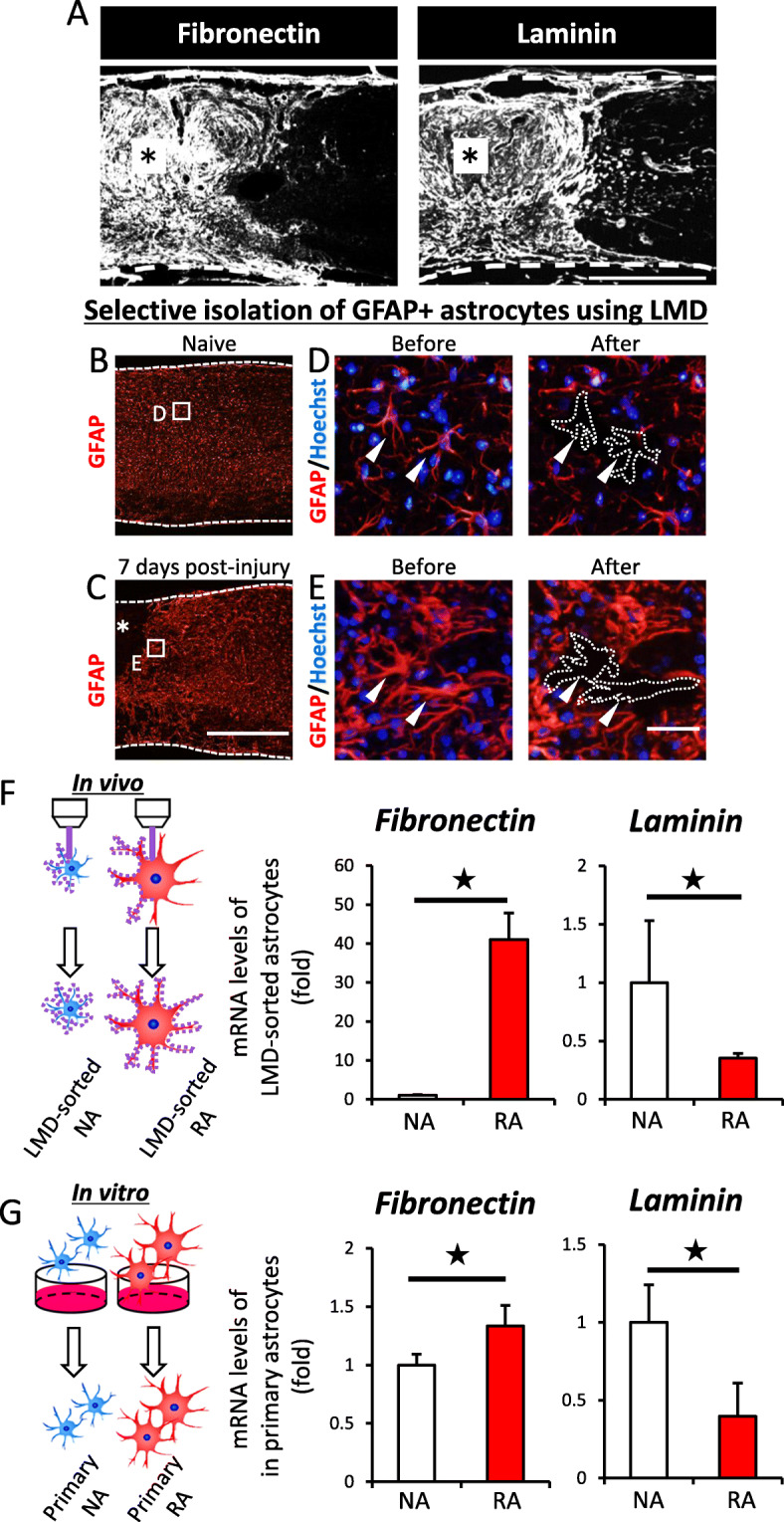
Reactive astrocytes expressed fibronectin both in vitro and in vivo. **a** Sagittal sections of chronically injured spinal cord. Asterisk indicates the lesion epicenter. Fibronectin, Laminin, white. Scale bar, 500 μm. **b** Sagittal section of naïve spinal cord. Magnification of the inset is shown in **d**. GFAP, red. **c** Sagittal section of injured spinal cord at 7 days post-injury. Magnification of the inset is shown in **e**. GFAP, red. Asterisk indicates the lesion epicenter. Scale bar, 500 μm. **d**, **e** GFAP-positive astrocytes (marked by white arrow-heads) were isolated marginally (surrounded area by white dots) by laser-captured microdissection (LMD). GFAP, red; Hoechst, blue. Scale bar, 20 μm. **f** Both NAs and RAs were isolated from spinal cord by LMD in vivo. Purple dots indicate the cutting line of the peri-lesional area. The mRNA expression of fibronectin and laminin, ligands of β1 integrin receptor, was analyzed by qPCR. Error bar indicates mean ± SEM. Star indicates statistical significance (*p* < 0.05). Wilcoxon’s rank-sum test. *n* = 3 per each group, triplicate. Fibronectin: *F* = 1.3 × 10^−6^. Laminin: *F* = 0.0385. **g** Both NAs and RAs were collected from primary cultures in vitro. The mRNA expression of fibronectin and laminin was analyzed by qPCR. Error bar indicates mean ± SEM. Star indicates statistical significance (*p* < 0.05). Wilcoxon’s rank-sum test. *n* = 3 per each group, triplicate. Fibronectin: *F* = 0.0432. Laminin: *F* = 0.0002

To clarify the certain ligand factor in RACM, we evaluated the lesional distribution of major ligands of β1 integrin receptor (β1R) other than collagen, such as fibronectin and laminin [20], other than collagen described in our previous report [5]. We found that these ligands were broadly expressed in and around the lesional area in the chronic phase of SCI (Figs. 1b and 4a). We thus speculated that the alteration of microglial polarization by β1Ab administration (Figs. 1, 2, and 3) was associated with these factors.

Next, to examine whether or not these factors were actually secreted by astrocytes in vivo after SCI, we selectively isolated naïve astrocytes (NAs) from naïve spinal cords and RAs from injured spinal cord at 7 dpi using LMD (Fig. 4b–e). Interestingly, the mRNA expression of fibronectin in RAs was significantly upregulated, whereas that of laminin was significantly downregulated compared to NAs (Fig. 4f). Consistent with the in vivo results, we confirmed that the mRNA expression of fibronectin in RAs was significantly upregulated, whereas that of laminin was significantly downregulated compared to NAs in vitro (Fig. 4g). These results suggested that fibronectin was a contributing factor to microglial inflammation induced by RACM stimulation (Fig. 3).

### In vivo and in vitro reactive astrocytes interact with microglia within glial scar via fibronectin

**Fig. 5:**
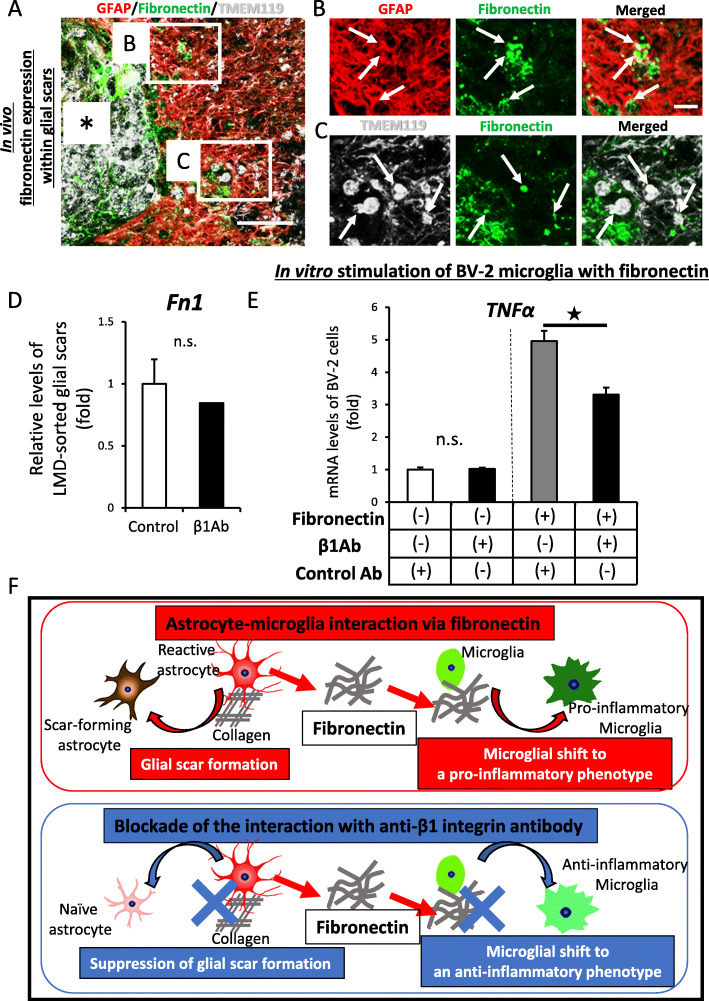
Fibronectin expressed by reactive astrocytes is associated with intercellular interaction between astrocytes and microglia in vivo. **a** Peri-lesional glial scar of the chronically injured spinal cord. Asterisk indicates the lesion epicenter. GFAP, red; Fibronectin, green; TMEM119, white. Scale bar, 200 μm. **b** Magnification of inset b in **a**. GFAP, red; Fibronectin, green. Scale bar, 100 μm. **c** Magnification of inset c in **a**. CD11b, white; Fibronectin, green. Scale bar, 100 μm. **d** The administration of anti-β1 integrin antibody had no effect on the mRNA expression of Fn1 within glial scars. The error bar indicates mean ± SEM. Star indicates statistical significance (*p* < 0.05). n.s., not significant. Wilcoxon’s rank-sum test. *n* = 4 per each group, duplicate. *F* = 0.192. **e** The TNFα mRNA expression of BV-2 cells after fibronectin stimulation with or without β1Ab pre-treatment. Error bar indicates mean ± SEM. Star indicates statistical significance (*p* < 0.05). Wilcoxon’s rank-sum test. *n* = 3 per each group, triplicate. **f** Our hypothesis of the novel glial scar pathology and therapeutic effects of anti-β1 integrin antibody. Fibronectin is suggested to be expressed by reactive astrocytes and recognized by the β1 integrin receptor in microglia. Microglia attain a pro-inflammatory phenotype by fibronectin. As previously reported, the antibody blocked the interaction between reactive astrocytes and collagen, leading to the suppression of glial scar formation. The present findings suggested that the antibody also blocked interaction between reactive astrocytes and fibronectin, leading to microglia polarization within the glial scar to an anti-inflammatory condition. These integrated effects of anti-β1 integrin antibody administration can modulate the glial scar pathology and improve the chronic microenvironment after SCI

To examine the protein expression of fibronectin in RAs in the chronic phase after SCI in vivo, we performed immunohistochemical analyses at the glial scar area in control Ab-treated mice. As shown in Fig. 5, we found that fibronectin protein was colocalized with both GFAP-positive astrocytes and TMEM119-positive microglia (Fig. 5a–c). In β1Ab-treated mice, we hardly detected any colocalization at the glial scar area. These results suggested that there might be interaction between astrocytes and microglia via fibronectin and that β1Ab might block this interaction.

Next, to clarify what happened to the fibronectin expression within the glial scar after β1Ab administration, we collected the area of the glial scar and analyzed the expression of fibronectin. The mRNA expression of fibronectin was not significantly changed by β1Ab administration (Fig. 5d), suggesting that β1Ab had no effect on the mRNA expression of fibronectin within the glial scar. Taking into account the results of fibronectin stimulation experiments in vitro, it was suggested that β1Ab administration could not downregulate the expression of fibronectin but able to inhibit the connection between fibronectin and microglia via β1 integrin receptors, which suppressed the pro-inflammatory effect of fibronectin.

To verify this, we stimulated BV-2 microglia by fibronectin and examined the gene expression of TNFα in vitro as previously described [21]. Consistent with the results of our RACM stimulation experiments, the mRNA expression of TNFα was upregulated in a fibronectin-dependent manner. In addition, this upregulation of TNFα was significantly suppressed by pre-treatment of β1Ab (Fig. 5e). These results suggested that BV-2 microglia were activated toward a pro-inflammatory phenotype by fibronectin in RACM and that β1Ab treatment significantly blocked the interaction between microglia and astrocytes via fibronectin.

Taken together, these findings indicate that fibronectin is expressed by RAs, mediates the interaction between RAs and microglia, and induces microglia toward a pro-inflammatory phenotype. β1Ab exerts not only a suppressing effect on glial scar formation but also an ameliorating effect on the pro-inflammatory condition by blocking intercellular interaction between RAs and microglia, leading to an improvement of the microenvironment after chronic SCI (Fig. 5f).

## Discussion

In the present study, we showed that β1Ab administration suppressed microglial inflammation and ameliorated the pathological microenvironment in the chronic phase after SCI. Fibronectin, a major ligand of β1R, was secreted by reactive astrocytes and significantly associated with microglial inflammation. These findings highlight the therapeutic effects of β1Ab administration as a modulator of chronic neuroinflammation as well as glial scar formation after SCI.

The cellular and extracellular components of the glial scar are known to interact with each other, inhibit the repairing processes by neural cells, and result in the formed scar becoming permanently undegradable after SCI [22]. Astrocytes were previously reported to be activated by microglia-derived factors [12, 23]. In the present study, we first demonstrated that microglia were conversely activated by an astrocyte-derived fibronectin. This result suggested the bidirectional and complex interaction between microglia and astrocytes within the glial scar. Microglia are also reported to interact with neurons [24] and oligodendrocytes [25], exerting both beneficial and harmful effects on SCI pathology [26, 27]. Although microglia have multifaceted roles after SCI and the cell-cell interactions remain unclear, the simple depletion of microglia was reported to fail to improve functional recovery after SCI [13, 28, 29]. In fact, when microglial activation was suppressed in the acute phase, beneficial responses such as DAMP clearance and reactive astrogliosis were reported to be disturbed [30]. In addition, when microglial activation was suppressed in the chronic phase, the regenerative and anti-inflammatory responses were reported to be disturbed [31]. Since these previous reports strongly suggested that microglial activation was phase-dependent and bifacial, blocking only the harmful interaction between microglia and astrocytes at the appropriate timing appears to be the key to improving the pathological environment after SCI.

In the present study, we induced primary astrocytes to differentiate into RAs using IL-6/IL-6R, as previously described [5, 32]. However, the P3 astrocytes used for cell culture are immature and likely do not have the same properties as adult mature astrocytes in the spinal cord after injury [33]. Some researchers previously reported an inability to reproduce the properties of astrocytes in vitro [34, 35]. IL-6/IL-6R used in our study is known to induce differentiation into RAs via the gp130 and JAK/STAT signaling pathways [6, 36]. In this study, we confirmed that both in vivo RAs after SCI and in vitro RAs with IL-6/IL-6R similarly expressed fibronectin (Additional file 6). Even though our in vitro RAs were unable to completely mimic in vivo RAs found in nature, our results suggested that our RACM consisted of fibronectin and that RAs interacted with microglia via fibronectin both in vivo and in vitro.

Fibronectin is an ECM that is increased in SCI lesions. The pathological role of fibronectin after SCI has been described as a major extracellular component of fibrous scarring in the lesion epicenter [15, 37]. However, in the present study, we first reported the role of fibronectin as a mediator of intercellular interaction within the glial scar. In general, fibronectin is produced by hepatocyte and exists in the circulation. However, after SCI, fibronectin not only extravasates through the disrupted blood-spinal cord barrier but is also de novo produced by astrocytes [38–40]. Recently, in the pathology of multiple sclerosis, the deposition of fibronectin in a lesion was reported to induce a pro-inflammatory microenvironment [41]. Fibronectin is also reported to induce the TNFα expression in mononuclear leukocytes in a dose-dependent manner [21]. In addition, fibronectin in the spinal cord is reported to activate microglia toward a pro-inflammatory polarization via β1R [42]. In the present study, we showed that fibronectin was secreted by RAs and activated microglia toward a pro-inflammatory condition by binding with β1R in chronic SCI pathology.

One limitation associated with this study was that mass spectrometry was not performed in order to examine what proteins existed and activated microglia in RACM. However, given the report that the culture supernatant of rat astrocytes contains fibronectin [39] and that the mRNA expression of fibronectin was upregulated in astrocytes in our in vivo and in vitro experiments, fibronectin likely existed in RACM and induced pro-inflammatory microglia-mediated neuroinflammation. In addition, there were likely some other molecules or ECM components in the RACM that exacerbated the effect of fibronectin on BV-2 cells. In the present study, although the fibronectin mRNA expression of the in vitro RAs was modestly upregulated (Fig. 4g), RACM stimulation strongly upregulated the TNFα mRNA expression of BV-2 microglia (Fig. 3c). The significant changes in each experiment demonstrated that (1) astrocytes significantly upregulated the mRNA expression of pro-inflammatory proteins (including fibronectin) after SCI, (2) the microglial mRNA expression of TNFα was significantly upregulated by some proteins released by RA (including fibronectin), and (3) β1Ab was able to significantly block the interaction between microglia and some proteins released by RAs.

Besides the mediator of inflammatory response, fibronectin is reported to enhance the phagocytic function in leukocytes, microglia, by binding to β3 integrin [43]. Their phagocytosis is crucial in the pathophysiology of the CNS, as Shichita et al. reported that Msr1 (C204) was associated with the resolution of chronic inflammation by enhancing DAMP clearance after brain infarction [44, 45]. In the present study, we also confirmed that fibronectin upregulated the mRNA expression of Msr1 in microglia. However with β1Ab pre-treatment, the microglial expression of Msr1 was upregulated after RACM stimulation, while it was downregulated after fibronectin stimulation alone (Fig. 3c, Additional file 7). This difference might be due to the existence of various proteins other than fibronectin in RACM. Given that fibronectin binds to both β3- and β1-integrin receptors [46], the blocking effect for β1R by β1Ab treatment may enhance the binding with β3 integrin and phagocytosis of various proteins within RACM [46, 47]. Consistent with the results of the RACM stimulation experiments (Fig. 3c), the mRNA expression of Msr1 within the glial scar was significantly upregulated by β1Ab (Fig. 2c). These results suggested that the RACM stimulation experiments in the present study were suitable for evaluating the in vivo intercellular interaction between microglia and astrocytes. Finally, we confirmed that the microglial expression of TNFα was not suppressed in a dose-dependent manner by β1Ab treatment (Additional file 8). In conclusion, the indirect blockade of the interaction between microglia and astrocytes via fibronectin promoted CNS repair while limiting chronic neuroinflammation and accelerating the clearance of damage signals within the glial scar after SCI.

## Conclusions

Microglial inflammation was enhanced by RAs via the fibronectin/β1 integrin pathway within the glial scar after SCI. Our results suggested that β1Ab administration has therapeutic potential for ameliorating both glial scar formation and persistent neuroinflammation in the chronic phase after SCI.

## Supplementary Information

**Additional file 1: Figure S1.** The accuracy of our spinal cord injury model using IH impactor. The displacement is shown. n.s., not significant. Wilcoxon’s rank-sum test. n = 14–15 mice per group. F=0.671.

**Additional file 2: Figure S2.** The administration of anti-β1 integrin antibody in the sub-acute phase significantly downregulated the mRNA expression relative to astroglial activation and axonal regeneration compared to control antibody in injured spinal cord. Error bar indicates mean±SEM. ★ indicates statistical significance (p<0.05). n.s., not significant. Wilcoxon’s rank-sum test. n=4 per each group, duplicate. Gfap: F=7.7×10^-11^, Col1α1: F=0.509. Gap43: F=0.017. Arg1: F=0.0013.

**Additional file 3: Figure S3.** The administration of anti-β1 integrin antibody markedly downregulated the mRNA expression of TNFα and significantly upregulated the mRNA expression of Msr1 compared to control antibody in injured spinal cord. Error bar indicates mean±SEM. ★ indicates statistical significance (p<0.05). n.s., not significant. n.d., not detectable. Wilcoxon’s rank-sum test. n=4 per each group, duplicate. TNFα: F=0.0002. Il1b: F=0.0788. Msr1: F=0.0201.

**Additional file 4: Figure S4.** The administration of anti-β1 integrin antibody made relatively limited effects on microglial mRNA expressions. Error bar indicates mean±SEM. ★ indicates statistical significance (p<0.05). n.s., not significant. Wilcoxon’s rank-sum test. n=4 per each group, duplicate. Fcgr3(CD16): F=0.610. Fcgr2(CD32): F=0.244. CD86: F=0.2211. Chil3(YM1): F=0.021. Ccl1: F=0.426. Ccl8: F=0.421. Ccl17: F=0.274. Ccl22:F=0.650. Ccl24: F=0.141. Cxcl1: F=0.993. Cxcl2: F=0.168. Cxcl3: F=0.275. Cxcl9: F=0.111. Cxcl10: F=0.112. Cxcl11: F=0.100. Cxcl13: F=0.923. Il15: F=0.663. Il23a: F=0.136. Tgfb1: F=0.437. Igf1: F=0.610. Pdgfa: F=0.360. Gdgfb: F=0.907.

**Additional file 5: Figure S5.** BV-2 microglial cells used in our *in vitro* experiments expressed TMEM119, which is a specific protein of microglia. CD11b: green, TMEM119: red, Hoechst: blue. Scale bar: 20 μm.

**Additional file 6: Figure S6.** Both *in vivo* and *in vitro* reactive astrocytes similarly expressed each transcript variant of fibronectin. Although the fibronectin gene transcript had multiple sites of alternative splicing [48], the heatmap shows that the mRNA expression profile of transcript variants 1 to 7 is upregulated in reactive astrocytes of *in vivo* LMD-sorted and *in vitro* primary astrocytes.

**Additional file 7: Figure S7.** The Msr1 mRNA expression of BV-2 cells after fibronectin stimulation with or without β1Ab pre-treatment. Error bar indicates mean±SEM. ★ indicates statistical significance (p<0.05). Wilcoxon’s rank-sum test. n=3 per each group, triplicate.

**Additional file 8: Figure S8.** The effects of β1Ab treatment blocking microglia inflammation did not depend on the dose of β1Ab. (-) indicates without anti-β1 antibody. ×10 indicates a 10-fold dose of β1Ab. n.s., not significant. Dunnett’s test in comparison to the control group (RACM×1, β1Ab(-)).

## Data Availability

All data generated or analyzed during this study are included in this published article and its supplementary information files.

## Abbreviations

SCI: Spinal cord injury
TNF: Tumor necrosis factor
Msr: Macrophage scavenger receptor
GFAP: Glial fibrillary acidic protein
β1Ab: Anti-β1 integrin antibody
β1R: β1 integrin receptor
NA: Naïve astrocyte
RA: Reactive astrocyte
RACM: Conditioned medium of reactive astrocytes
