# Supplementary figures and images for: Microglial inflammation after chronic spinal cord injury is enhanced by reactive astrocytes via the fibronectin/β1 integrin pathway
Source: J Neuroinflammation. 2021 Jan 6;18:12. doi: 10.1186/s12974-020-02059-x (PMC7789752; doi:10.1186/s12974-020-02059-x)

## Slide 1
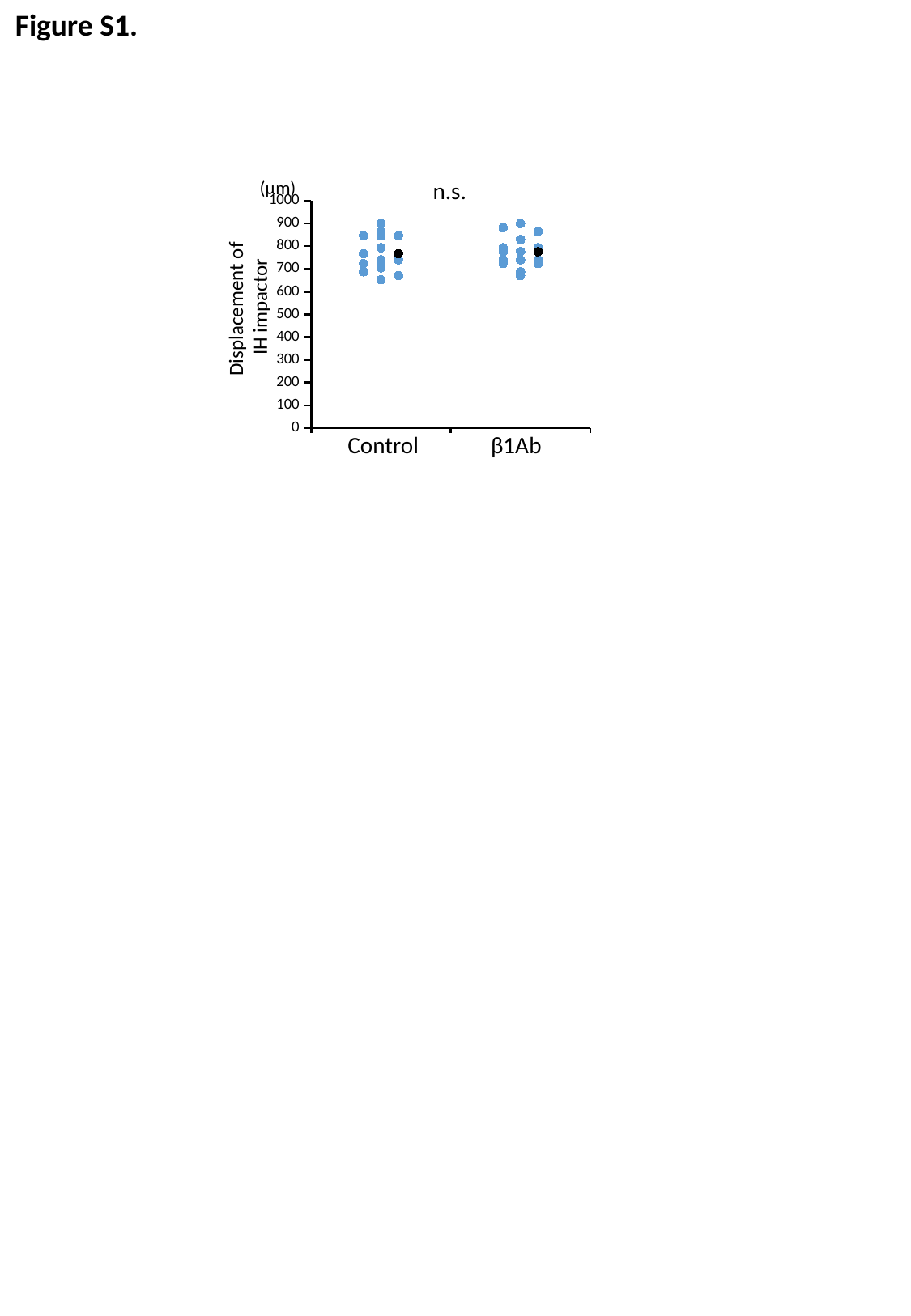

Figure S1.
(µm)
n.s.
### Chart
| Category | |
|---|---|Displacement of
 IH impactor
β1Ab
Control

Supplement: Supplementary file 1 — Additional file 1: Figure S1. The accuracy of our spinal cord injury model using IH impactor. The displacement is shown. n.s., not significant. Wilcoxon’s rank-sum test. n = 14–15 mice per group. F=0.671. [file 12974_2020_2059_MOESM1_ESM.pptx]

## Slide 1
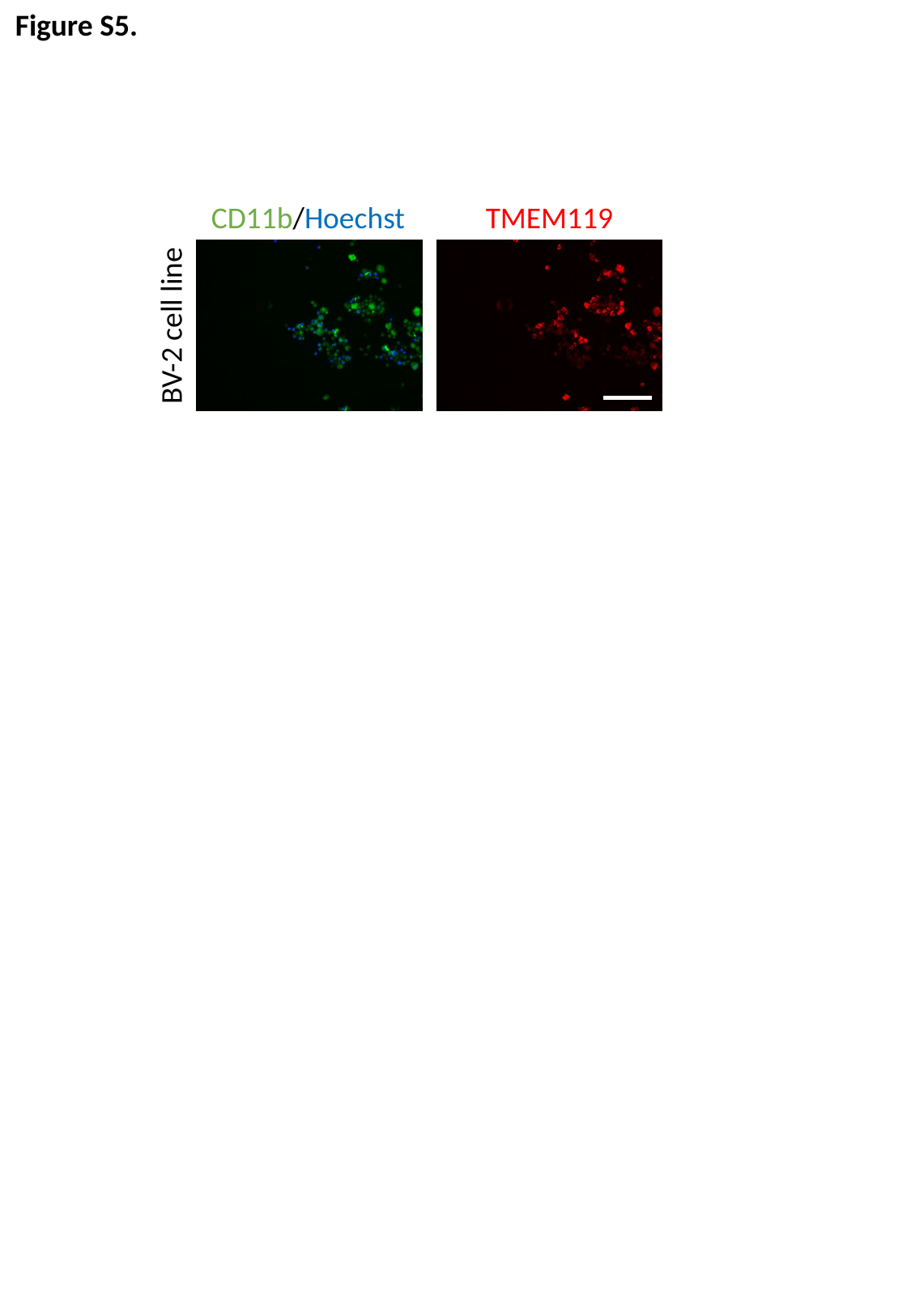

Figure S5.
CD11b/Hoechst
TMEM119
BV-2 cell line

Supplement: Supplementary file 5 — Additional file 5: Figure S5. BV-2 microglial cells used in our in vitro experiments expressed TMEM119, which is a specific protein of microglia. CD11b: green, TMEM119: red, Hoechst: blue. Scale bar: 20 μm. [file 12974_2020_2059_MOESM5_ESM.pptx]
