# Supplementary material for: Microglial inflammation after chronic spinal cord injury is enhanced by reactive astrocytes via the fibronectin/β1 integrin pathway
Source: J Neuroinflammation. 2021 Jan 6;18:12. doi: 10.1186/s12974-020-02059-x (PMC7789752; doi:10.1186/s12974-020-02059-x)
Supplement: Supplementary file 2 — Additional file 2: Figure S2. The administration of anti-β1 integrin antibody in the sub-acute phase significantly downregulated the mRNA expression relative to astroglial activation and axonal regeneration compared to control antibody in injured spinal cord. Error bar indicates mean±SEM. ★ indicates statistical significance (p<0.05). n.s., not significant. Wilcoxon’s rank-sum test. n=4 per each group, duplicate. Gfap: F=7.7×10^-11^, Col1α1: F=0.509. Gap43: F=0.017. Arg1: F=0.0013. [file 12974_2020_2059_MOESM2_ESM.pptx]

## Slide 1
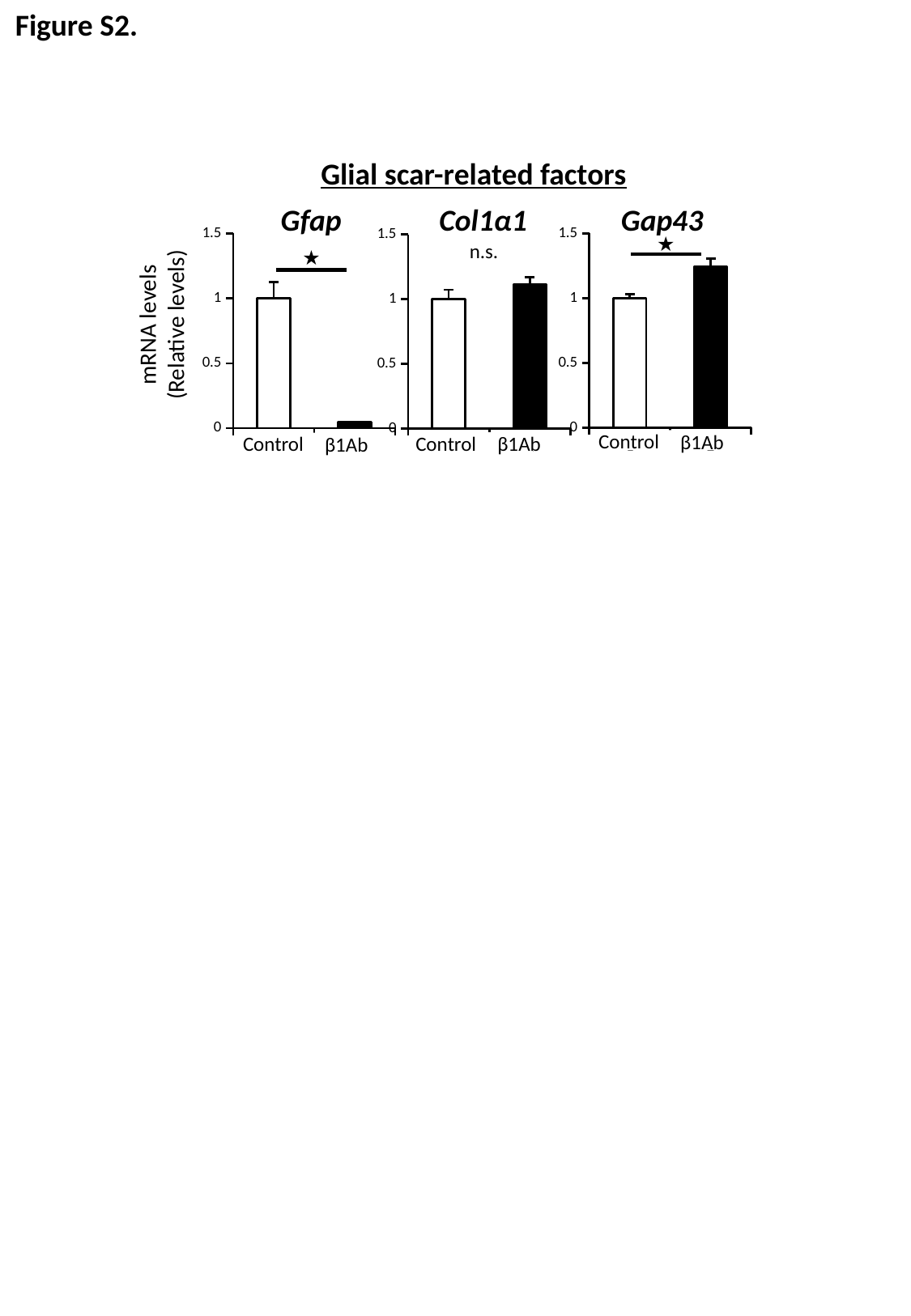

Figure S2.
Glial scar-related factors
Gfap
Col1α1
Gap43
### Chart
| Category | |
|---|---|
### Chart
| Category | |
|---|---|
### Chart
| Category | |
|---|---|★
n.s.
★
mRNA levels
(Relative levels)
Control
β1Ab
Control
Control
β1Ab
β1Ab
