# Supplementary material for: Microglial inflammation after chronic spinal cord injury is enhanced by reactive astrocytes via the fibronectin/β1 integrin pathway
Source: J Neuroinflammation. 2021 Jan 6;18:12. doi: 10.1186/s12974-020-02059-x (PMC7789752; doi:10.1186/s12974-020-02059-x)
Supplement: Supplementary file 3 — Additional file 3: Figure S3. The administration of anti-β1 integrin antibody markedly downregulated the mRNA expression of TNFα and significantly upregulated the mRNA expression of Msr1 compared to control antibody in injured spinal cord. Error bar indicates mean±SEM. ★ indicates statistical significance (p<0.05). n.s., not significant. n.d., not detectable. Wilcoxon’s rank-sum test. n=4 per each group, duplicate. TNFα: F=0.0002. Il1b: F=0.0788. Msr1: F=0.0201. [file 12974_2020_2059_MOESM3_ESM.pptx]

## Slide 1
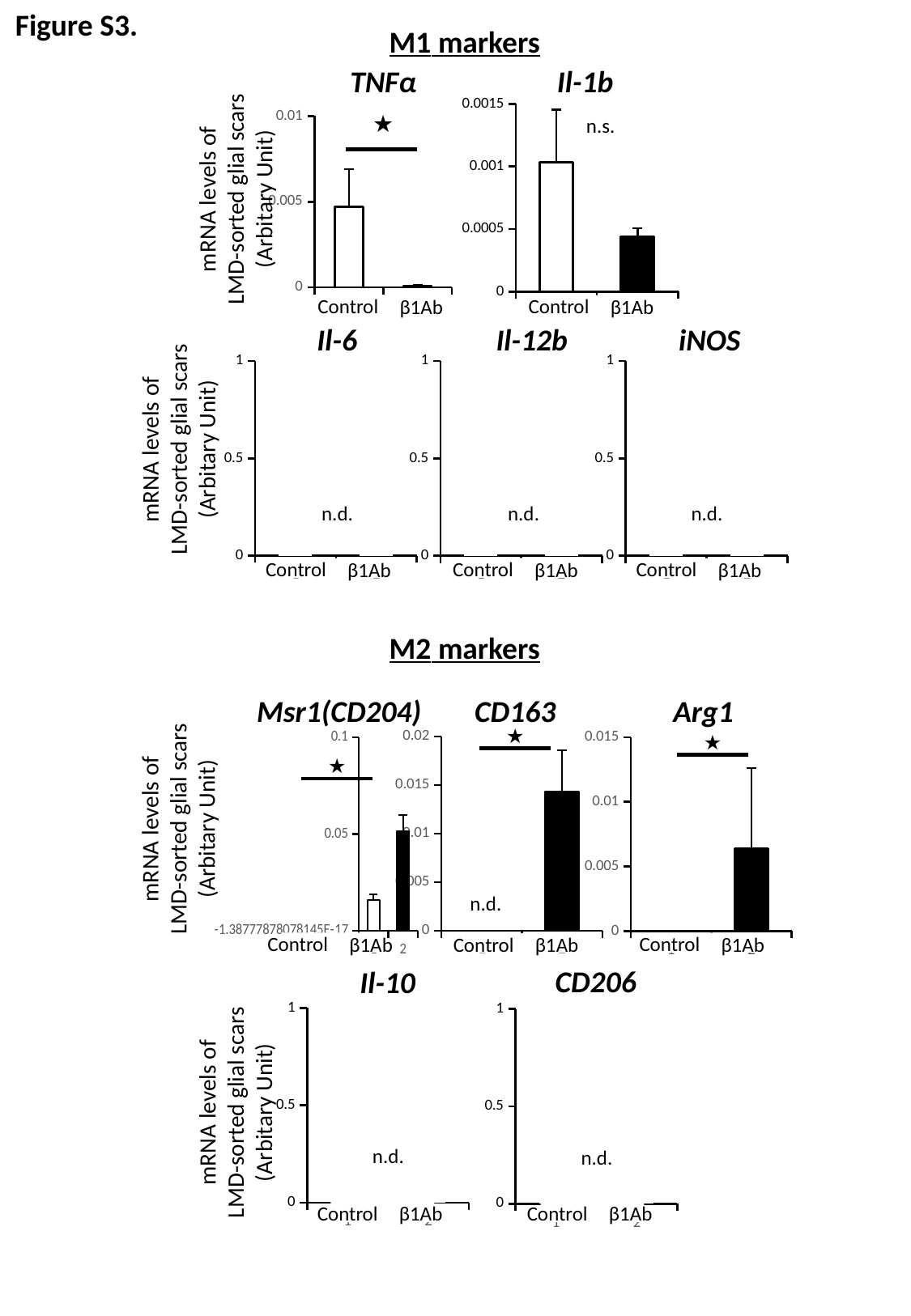

Figure S3.
M1 markers
Il-1b
TNFα
### Chart
| Category | |
|---|---|★
### Chart
| Category | |
|---|---|n.s.
mRNA levels of
LMD-sorted glial scars
(Arbitary Unit)
Control
Control
β1Ab
β1Ab
iNOS
Il-12b
Il-6
### Chart
| Category | |
|---|---|
### Chart
| Category | |
|---|---|
### Chart
| Category | |
|---|---|mRNA levels of
LMD-sorted glial scars
(Arbitary Unit)
n.d.
n.d.
n.d.
Control
Control
Control
β1Ab
β1Ab
β1Ab
M2 markers
CD163
Msr1(CD204)
Arg1
★
★
### Chart
| Category | |
|---|---|
### Chart
| Category | |
|---|---|
### Chart
| Category | |
|---|---|★
mRNA levels of
LMD-sorted glial scars
(Arbitary Unit)
n.d.
Control
Control
β1Ab
β1Ab
Control
β1Ab
CD206
Il-10
### Chart
| Category | |
|---|---|
### Chart
| Category | |
|---|---|mRNA levels of
LMD-sorted glial scars
(Arbitary Unit)
n.d.
n.d.
Control
Control
β1Ab
β1Ab
