# Supplementary material for: Microglial inflammation after chronic spinal cord injury is enhanced by reactive astrocytes via the fibronectin/β1 integrin pathway
Source: J Neuroinflammation. 2021 Jan 6;18:12. doi: 10.1186/s12974-020-02059-x (PMC7789752; doi:10.1186/s12974-020-02059-x)
Supplement: Supplementary file 4 — Additional file 4: Figure S4. The administration of anti-β1 integrin antibody made relatively limited effects on microglial mRNA expressions. Error bar indicates mean±SEM. ★ indicates statistical significance (p<0.05). n.s., not significant. Wilcoxon’s rank-sum test. n=4 per each group, duplicate. Fcgr3(CD16): F=0.610. Fcgr2(CD32): F=0.244. CD86: F=0.2211. Chil3(YM1): F=0.021. Ccl1: F=0.426. Ccl8: F=0.421. Ccl17: F=0.274. Ccl22:F=0.650. Ccl24: F=0.141. Cxcl1: F=0.993. Cxcl2: F=0.168. Cxcl3: F=0.275. Cxcl9: F=0.111. Cxcl10: F=0.112. Cxcl11: F=0.100. Cxcl13: F=0.923. Il15: F=0.663. Il23a: F=0.136. Tgfb1: F=0.437. Igf1: F=0.610. Pdgfa: F=0.360. Gdgfb: F=0.907. [file 12974_2020_2059_MOESM4_ESM.pptx]

## Slide 1
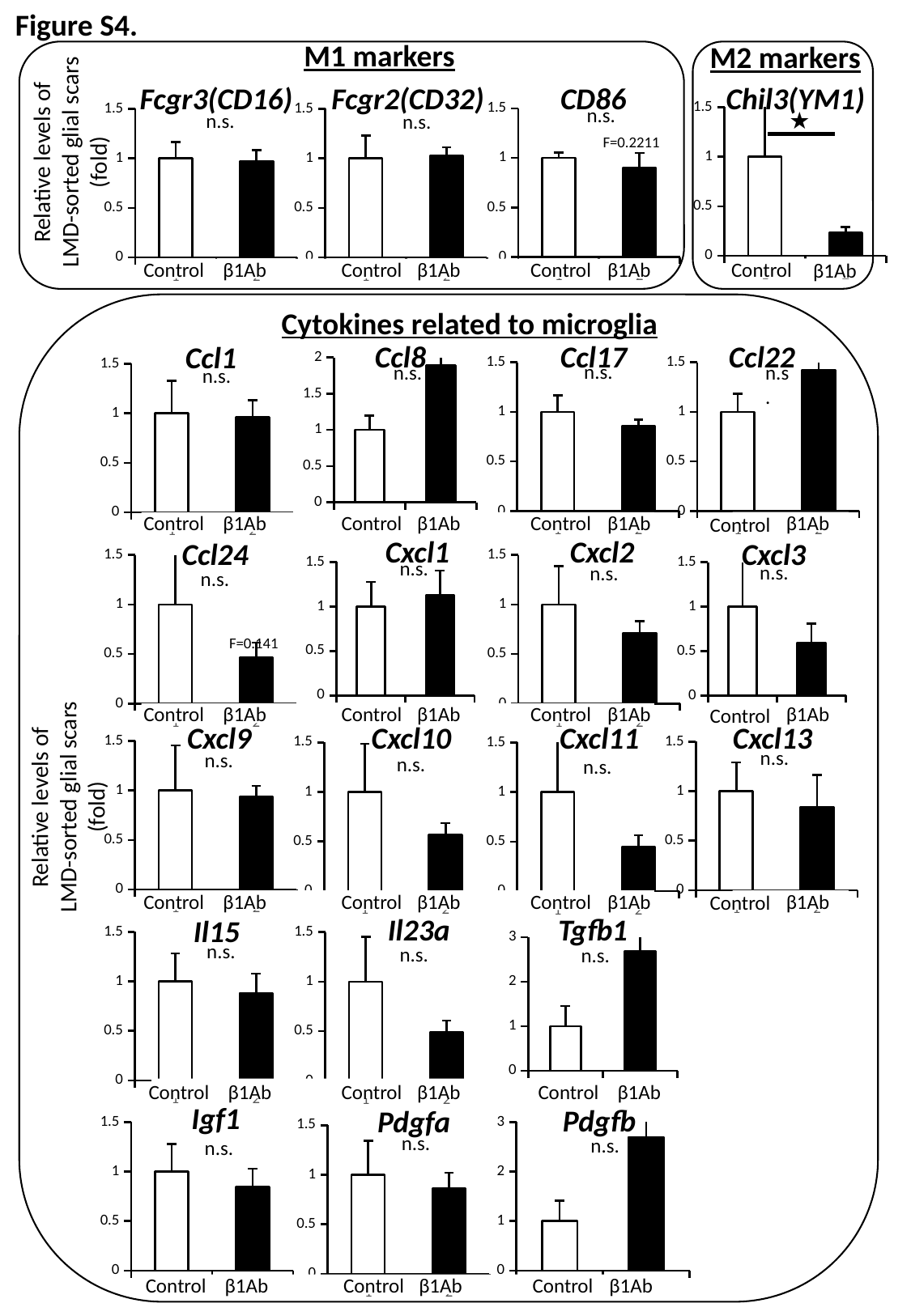

Figure S4.
M1 markers
M2 markers
Fcgr3(CD16)
Fcgr2(CD32)
CD86
Chil3(YM1)
### Chart
| Category | |
|---|---|n.s.
### Chart
| Category | |
|---|---|
### Chart
| Category | |
|---|---|
### Chart
| Category | |
|---|---|★
n.s.
n.s.
Relative levels of
LMD-sorted glial scars
(fold)
F=0.2211
Control
β1Ab
Control
β1Ab
Control
β1Ab
Control
β1Ab
Cytokines related to microglia
Ccl8
Ccl17
Ccl22
Ccl1
### Chart
| Category | |
|---|---|
### Chart
| Category | |
|---|---|
### Chart
| Category | |
|---|---|
### Chart
| Category | |
|---|---|n.s.
n.s.
n.s.
n.s.
Control
β1Ab
Control
β1Ab
Control
β1Ab
β1Ab
Control
Cxcl1
Cxcl2
Cxcl3
Ccl24
### Chart
| Category | |
|---|---|
### Chart
| Category | |
|---|---|
### Chart
| Category | |
|---|---|n.s.
### Chart
| Category | |
|---|---|n.s.
n.s.
n.s.
F=0.141
Control
β1Ab
Control
β1Ab
Control
β1Ab
β1Ab
Control
Cxcl9
Cxcl10
Cxcl11
Cxcl13
### Chart
| Category | |
|---|---|
### Chart
| Category | |
|---|---|
### Chart
| Category | |
|---|---|
### Chart
| Category | |
|---|---|n.s.
n.s.
n.s.
n.s.
Relative levels of
LMD-sorted glial scars
(fold)
Control
β1Ab
Control
β1Ab
Control
β1Ab
β1Ab
Control
Il23a
Tgfb1
Il15
### Chart
| Category | |
|---|---|
### Chart
| Category | |
|---|---|
### Chart
| Category | |
|---|---|n.s.
n.s.
n.s.
Control
β1Ab
Control
β1Ab
Control
β1Ab
Igf1
Pdgfb
Pdgfa
### Chart
| Category | |
|---|---|
### Chart
| Category | |
|---|---|
### Chart
| Category | |
|---|---|n.s.
n.s.
n.s.
Control
β1Ab
Control
β1Ab
Control
β1Ab
