# Supplementary material for: Microglial inflammation after chronic spinal cord injury is enhanced by reactive astrocytes via the fibronectin/β1 integrin pathway
Source: J Neuroinflammation. 2021 Jan 6;18:12. doi: 10.1186/s12974-020-02059-x (PMC7789752; doi:10.1186/s12974-020-02059-x)
Supplement: Supplementary file 6 — Additional file 6: Figure S6. Both in vivo and in vitro reactive astrocytes similarly expressed each transcript variant of fibronectin. Although the fibronectin gene transcript had multiple sites of alternative splicing [48], the heatmap shows that the mRNA expression profile of transcript variants 1 to 7 is upregulated in reactive astrocytes of in vivo LMD-sorted and in vitro primary astrocytes. [file 12974_2020_2059_MOESM6_ESM.pptx]

## Slide 1
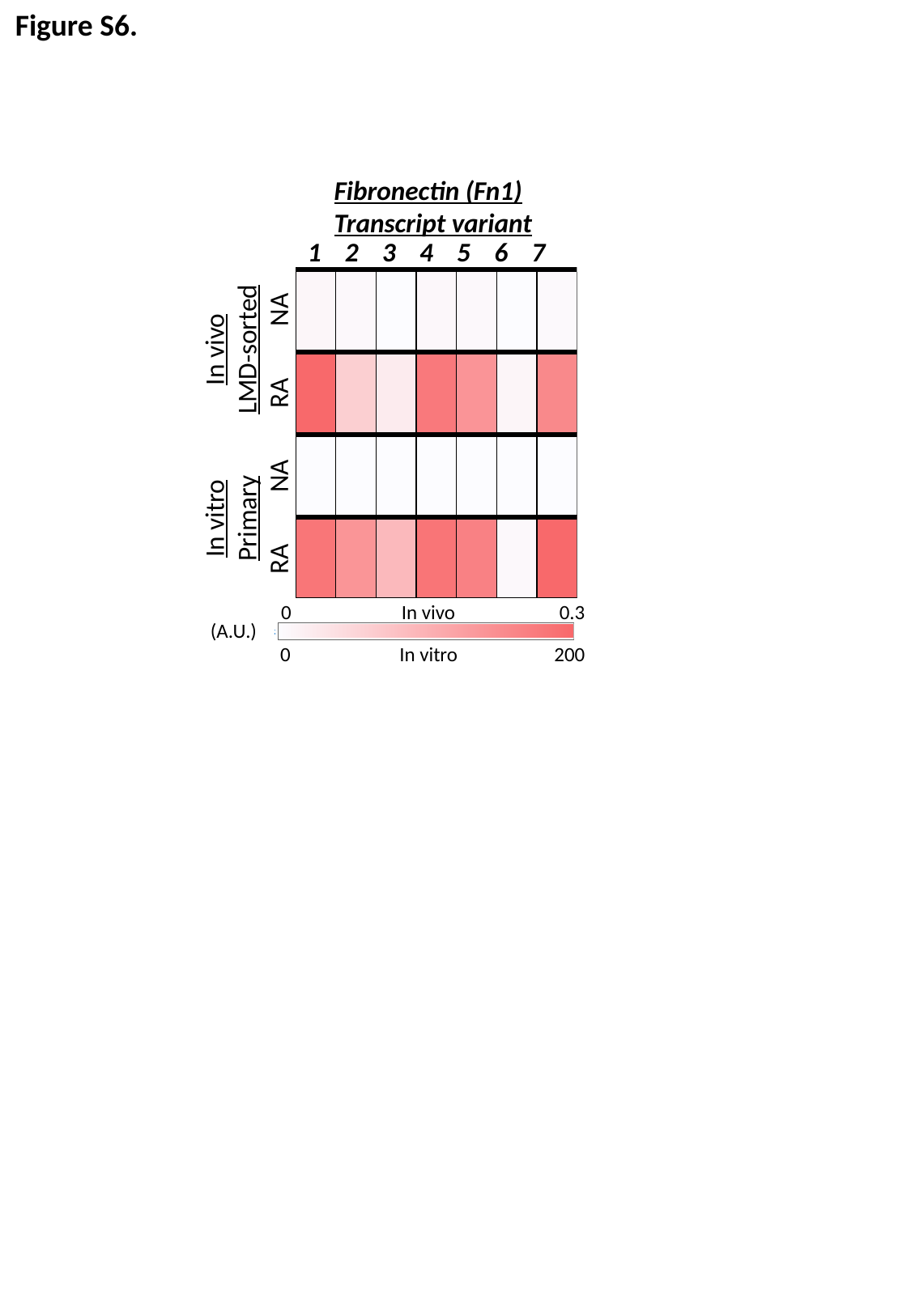

Figure S6.
Fibronectin (Fn1)
Transcript variant
1
2
3
4
5
6
7
NA
In vivo
LMD-sorted
RA
NA
In vitro
Primary
RA
0
In vivo
0.3
(A.U.)
0
In vitro
200
