# Supplementary material for: Microglial inflammation after chronic spinal cord injury is enhanced by reactive astrocytes via the fibronectin/β1 integrin pathway
Source: J Neuroinflammation. 2021 Jan 6;18:12. doi: 10.1186/s12974-020-02059-x (PMC7789752; doi:10.1186/s12974-020-02059-x)
Supplement: Supplementary file 7 — Additional file 7: Figure S7. The Msr1 mRNA expression of BV-2 cells after fibronectin stimulation with or without β1Ab pre-treatment. Error bar indicates mean±SEM. ★ indicates statistical significance (p<0.05). Wilcoxon’s rank-sum test. n=3 per each group, triplicate. [file 12974_2020_2059_MOESM7_ESM.pptx]

## Slide 1
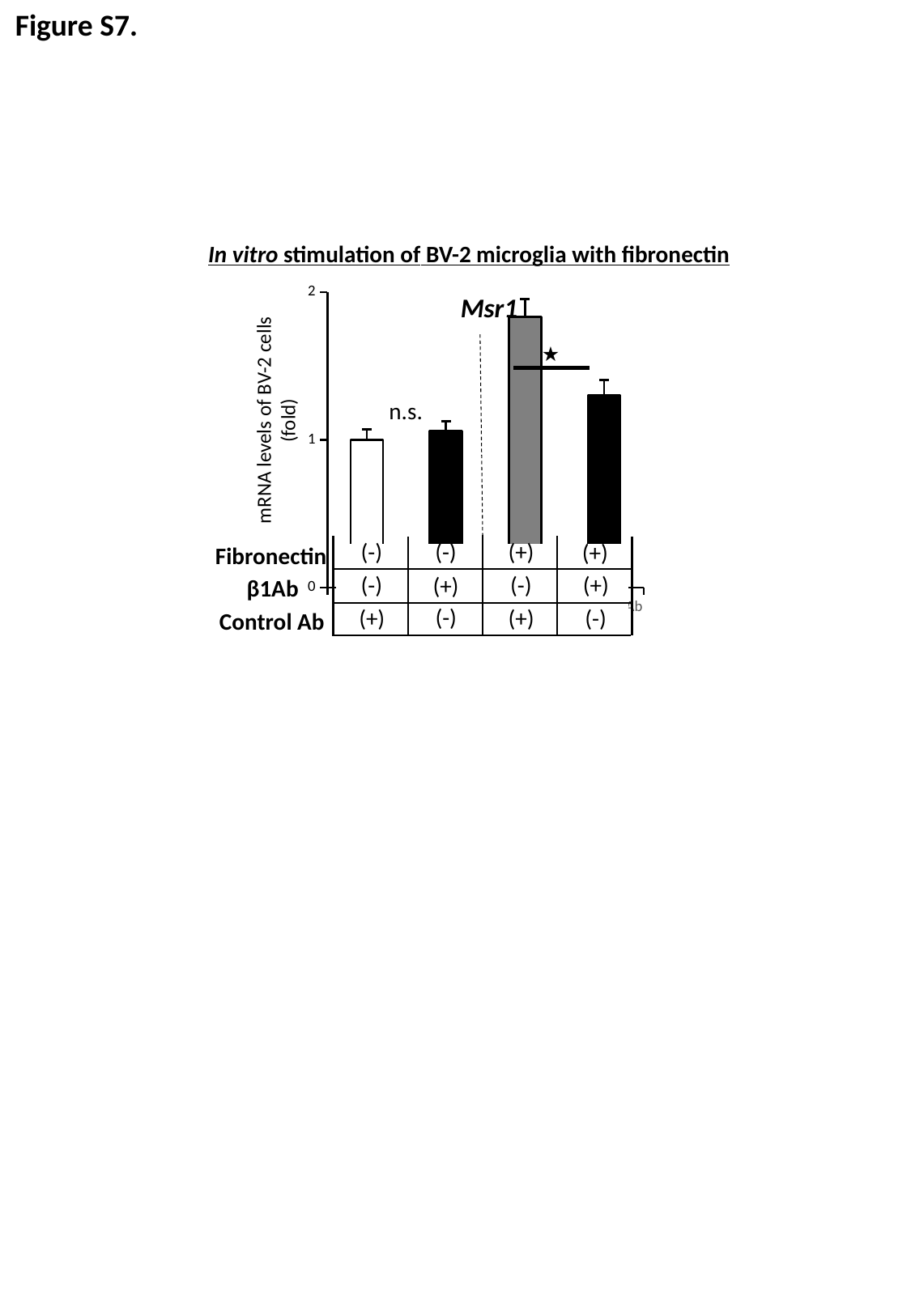

Figure S7.
In vitro stimulation of BV-2 microglia with fibronectin
### Chart
| Category | |
|---|---|
| Control | 1.0 |
| Control+Ab | 1.0599908294054865 |
| Fn1x100 | 1.8317770272575986 |
| Fn1x100+Ab | 1.2998187714958092 |Msr1
★
mRNA levels of BV-2 cells
(fold)
n.s.
(-)
(-)
(+)
(+)
Fibronectin
(-)
(-)
(+)
(+)
β1Ab
(-)
(+)
(+)
(-)
Control Ab
