# Supplementary material for: Microglial inflammation after chronic spinal cord injury is enhanced by reactive astrocytes via the fibronectin/β1 integrin pathway
Source: J Neuroinflammation. 2021 Jan 6;18:12. doi: 10.1186/s12974-020-02059-x (PMC7789752; doi:10.1186/s12974-020-02059-x)
Supplement: Supplementary file 8 — Additional file 8: Figure S8. The effects of β1Ab treatment blocking microglia inflammation did not depend on the dose of β1Ab. (-) indicates without anti-β1 antibody. ×10 indicates a 10-fold dose of β1Ab. n.s., not significant. Dunnett’s test in comparison to the control group (RACM×1, β1Ab(-)). [file 12974_2020_2059_MOESM8_ESM.pptx]

## Slide 1
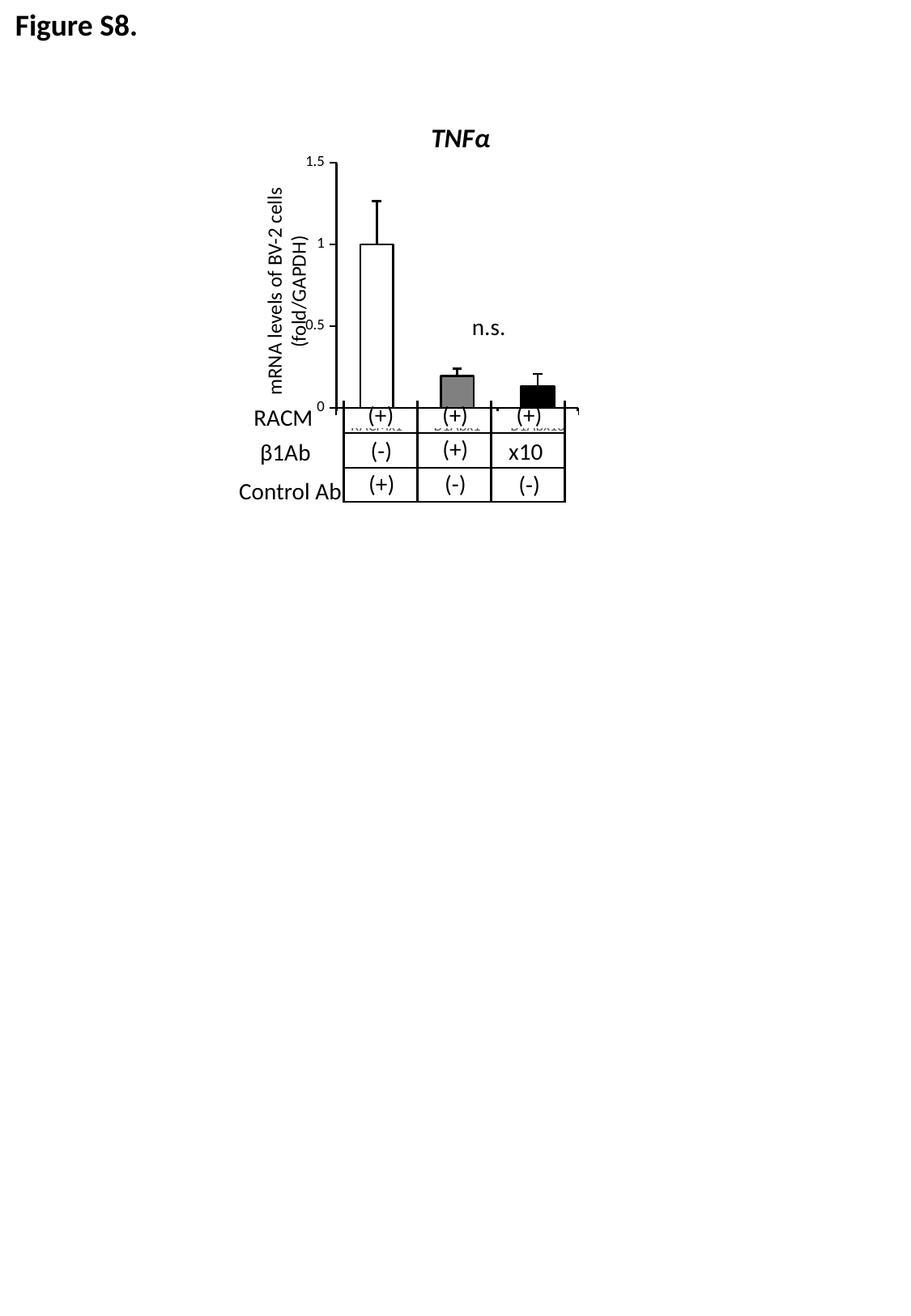

Figure S8.
TNFα
### Chart
| Category | |
|---|---|
| RACMx1 | 1.0 |
| B1Abx1 | 0.19324 |
| B1Abx10 | 0.13235 |mRNA levels of BV-2 cells
(fold/GAPDH)
n.s.
(+)
(+)
(+)
RACM
(+)
(-)
x10
β1Ab
(+)
(-)
(-)
Control Ab
